# Work-related outcomes in randomised placebo-controlled pain trials: a systematic review and meta-analysis

**DOI:** 10.1186/1745-6673-9-25

**Published:** 2014-07-15

**Authors:** Ingmar Wolf, Tim Friede, Ernst Hallier, Sebastian Straube

**Affiliations:** 1 Department of Medical Statistics, University Medical Center Göttingen, Humboldtallee 32, 37073 Göttingen, Germany; 2 Institute of Occupational, Social and Environmental Medicine, University Medical Center Göttingen, Waldweg 37 B, 37073 Göttingen, Germany

**Keywords:** Chronic painful conditions, Rheumatic diseases, Randomised controlled trials, Work-related outcomes

## Abstract

**Background:**

Chronic painful conditions have an important influence on the ability to work. Work-related outcomes, however, are not commonly reported in publications on trials investigating the treatment of chronic painful conditions. We aim to provide an overview of the reporting of work-related outcomes in such trials and investigate the relationship between work-related outcomes and pain outcomes.

**Methods:**

We conducted a systematic literature search in PubMed with the aim of identifying randomised placebo-controlled clinical trials investigating treatments for chronic painful conditions or rheumatic diseases that also reported on work-related outcomes. Methodological study quality was assessed with the Oxford Quality Scale (OQS). Meta-analyses were conducted for the outcomes of interference with work and number of patients with at least 30% reduction in pain intensity (30% pain responders). The correlation between work-related and pain outcomes was investigated with regression analyses.

**Results:**

We included 31 publications reporting on 27 datasets from randomised placebo-controlled trials (with a total of 11,434 study participants) conducted in chronic painful or rheumatic diseases and reporting on work-related outcomes. These 31 publications make up only about 0.2% of all publications on randomised placebo-controlled trials in such conditions. The methodological quality of the included studies was high; only nine studies scored less than four (out of a maximum five) points on the OQS. Sixteen different work-related outcomes were reported on in the studies. Of 25 studies testing for the statistical significance of changes in work-related outcomes over the course of the trials, 14 (56%) reported a significant improvement; the others reported non-significant changes. Eight studies reported data on both interference with work and 30% pain responders: meta-analyses demonstrated similar, statistically significant improvements in both these outcomes with active therapy compared to placebo and regression analysis showed that these outcomes were correlated.

**Conclusions:**

Despite the importance of pain as a reason for decreased ability to work, work-related outcomes are reported in substantially less than 1% of publications on placebo-controlled trials in chronic painful and rheumatic diseases. Work-related outcomes and pain responder outcomes are closely related.

## Background

Chronic painful conditions are very common as a recent systematic review of prevalence studies has demonstrated [1]. For example, a large survey found that about one fifth of European adults suffered from pain of at least six months’ duration and a third of those pain sufferers had severe pain [2]. Patients affected by a chronic painful disease experience the adverse effects of their condition on a number of domains of life, including work. In different studies 13% to 76% of chronic pain patients experienced loss of employment or were unable to undertake employment [1]. Those with moderate and, especially, severe pain are particularly affected [3]. Targeting inability to work and interference with work due to chronic pain therefore is important both from an individual as well as a societal, economic, perspective. It would be informative to know how work ability is affected by common pain treatments.

However, work-related outcomes are not commonly reported in publications on randomised controlled trials investigating the treatment of chronic painful conditions or rheumatic diseases (where pain typically is a prominent symptom). Where such data have been analysed, there is good evidence that those patients experiencing substantial improvements in pain outcomes in the context of clinical trials also experience a substantial improvement in their ability to work [4]. The question is how generalisable this agreement between work-related and pain-related study outcomes is.

With this publication we aim to, firstly, provide an overview of the reporting of work-related outcomes in chronic pain trials and, secondly, investigate the relationship between work-related outcomes and pain-related outcomes across different studies. Because for pain intensity the reporting as ‘responder outcomes’, such as the proportion of study participants experiencing at least 30%, or 50%, reduction in pain intensity over the course of a trial (30% or 50% pain responders), is more informative than treatment group average data, we focus on pain responder outcomes; this is in agreement with recent guidance on performing systematic reviews in the chronic pain field [5].

## Methods

### Literature search, study quality assessment, and data extraction

**Figure 1:**
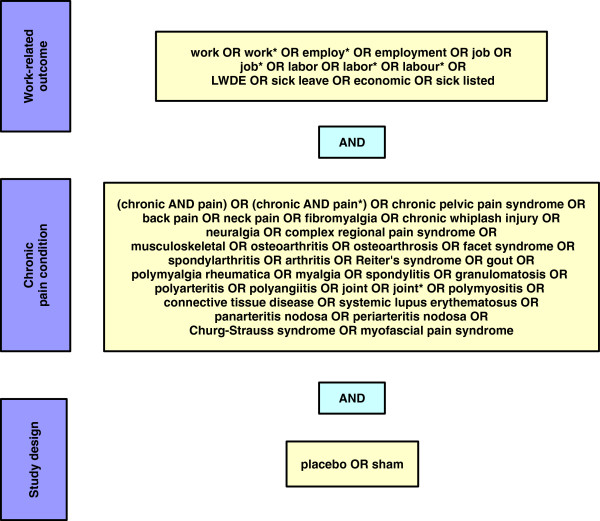
**Search strategy.** LWDE – lost workday equivalents.

We conducted a systematic literature search in Medline (PubMed) with the aim of identifying randomised controlled clinical trials investigating treatments for chronic painful conditions or rheumatic diseases that reported on any work-related study outcomes. We limited ourselves to placebo-controlled (or sham-controlled) studies to ensure some basic comparability between the studies and to be able to compare active treatments vs. placebo for work-related and pain-related study outcomes.Our search strategy is shown in Figure 1 and included search terms to identify work-related outcomes, search terms to identify chronic painful conditions or rheumatic diseases, and search terms to identify placebo or sham controlled studies. We did not activate any filters in PubMed but limited ourselves to articles published as full papers in English or German. The date of the last search was 13 June 2013. To estimate the total number of publications that could potentially have reported on work-related outcomes, we also performed our search without the work-related terms.

For inclusion in our systematic review studies needed to be single or double blind, use a placebo or sham control, include patients suffering from chronic pain (of at least 3 months’ duration) or with a rheumatic disease, and report on any work-related outcomes. We anticipated that a variety of work-related measures would be reported in the studies. In order to be inclusive we accepted any outcome measure that specifically addresses work or any aspect of work and were work-related effects were reported separately (i.e. not only as a summary score covering work and other domains of life). Studies investigating children or experimentally induced pain were excluded.

We assessed the methodological study quality with the Oxford Quality Scale, a standard and very widely used instrument for evaluating clinical trials that assesses the domains of randomisation, blinding and withdrawals/dropouts, and grades study quality on a scale of zero to five points [6].

Data were extracted on the publication details, the conditions studied, the treatments investigated, study size and duration, as well as work-related and pain-related study outcomes.

### Data analysis

We assessed means (and standard deviations [SDs]) of differences in work-related outcomes between trial beginning and end. If a study did not report SDs for mean differences between baseline and end of study values, SDs were estimated from p-values from hypothesis tests on the baseline to trial end differences. Studies reporting medians and interquartile ranges only had their data transformed to means and SDs by assuming the median to be the mean and the interquartile range to be 1.35 SDs.

Meta-analysis of data was performed with Review Manager (RevMan) [7]. Meta-analyses were conducted for the outcomes of ‘interference with work’ and 30% pain responders. Data for ‘interference with work’ were from component questions of the Brief Pain Inventory (question on pain interfering with normal work, including both work outside the home and housework), the Fibromyalgia Impact Questionnaire (question on how much pain or other symptoms of fibromyalgia interfered with the ability to do work, including housework) and the Sheehan Disability Scale (question on how much the symptoms have disrupted work or school work). These three questions were all assessed on scales of 0–10 points.

For meta-analyses the random effects model was used. If the between study heterogeneity was estimated to be zero (i.e. I^2^ = 0), the analysis is equivalent to a fixed effect meta-analysis.

The relationships between the outcomes of interference with work and 30%, or 50%, pain responders were investigated with regression analyses.

A further regression analysis was conducted for work-related and pain-related outcomes expressed as mean differences. In order to utilise all available data, which were expressed as different outcomes, we standardised the mean differences of the work-related and pain-related endpoints, as described by Hedges [8], before performing linear regression analysis.

All regression analyses were conducted in R [9]. The weights for the included studies were assigned according to the inverse variances of their work-related endpoints.

## Results

### Reporting of work-related outcomes

**Figure 2:**
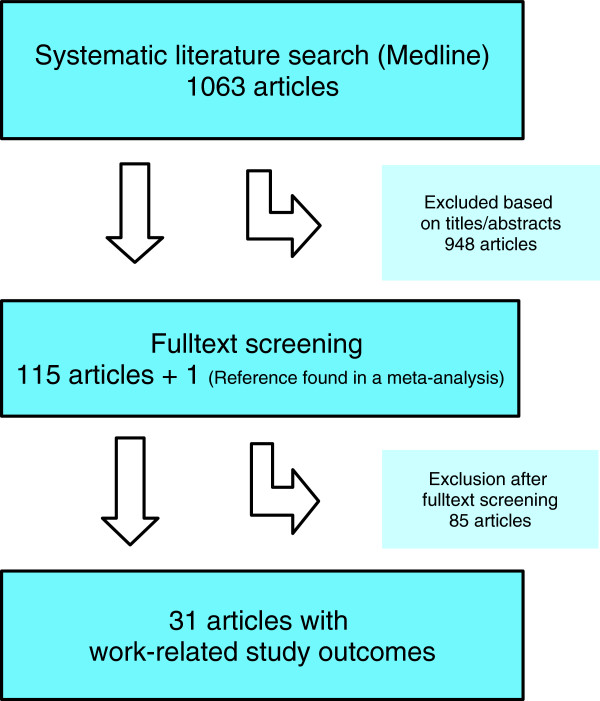
Study selection.

Our systematic literature search (Figure 2) yielded 1063 potentially relevant hits of which 948 were excluded as not relevant on the basis of study titles and abstracts. One hundred and fifteen remaining articles and one additional study from the references of a meta-analysis were examined as full texts. Eighty five of these full texts needed to be excluded. Additional file 1 details the excluded studies with reasons for their exclusion.

We identified as suitable for inclusion in our systematic review 31 publications [4,10-39] reporting on 27 datasets from randomised placebo-controlled trials in chronic painful diseases or rheumatic diseases (Table 1). Typically such a dataset corresponds to data from one clinical trial. Arnold et al. [12] and Bradley et al. [18] are meta-analyses that incorporated the same four clinical trials [11,20,40,41]. Two of these published work-related data [11,20]. In order to avoid counting the same patients more than once, all these studies were treated as one dataset. Bennett et al. [16] and Bennett et al. [17] were also treated as one dataset, because they both reported on the same trial. Furthermore, Straube et al. [4] reported an individual patient meta-analysis of work-related data from four trials but was counted as one dataset because the work-related data had not been published separately for those trials.

**Table 1:** Details of the included studies

**Data sets**	**Articles**	**Conditions**	**Active interventions**	**Patients**	**Work-related outcomes**	**Study duration (in weeks)**	**OQS score**
1	Albert et al. 2013 [10]	Chr. back pain	Amoxicillin–Clavulanate	162	Time lost from work	52	5
2	Baron et al. 2010 [14]	Chr. back pain	Pregabalin	217	WPAI	5	4
3	Carlsson & Sjölund 2001 [19]	Chr. back pain	Acupuncture, electroacupuncture	51	Employment status	32	5
4	Jarzem et al. 2005 [25]	Chr. back pain	TENS	350	McGill Work Scale	12	5
5	Lehmann et al. 1986 [29]	Chr. back pain	TENS, electroacupuncture	54	Employment status	3	4
6	Licciardone et al. 2003 [30]	Chr. back pain	Osteopathic manipulative treatment	91	Time lost from work	20	3
7	Skljarevski et al. 2009 [34]	Chr. back pain	Duloxetine	404	BPI-I	13	5
8	Skljarevski et al. 2010a [35]	Chr. back pain	Duloxetine	236	BPI-I, WPAI	13	4
9	Skljarevski et al. 2010b [36]	Chr. back pain	Duloxetine	401	BPI-I, WPAI	12	3
10	He et al. 2005 [24]	Chr. neck pain	Body acupuncture + body electrostimulation + ear acupressure	24	Activity impairment at work	4	2
11	Manchikanti et al. 2010 [31]	Chr. neck pain	Bupivacaine and steroid injection	120	Employability, employment status	96	5
12	Bennett et al. 2003 + 2005 [16,17]	Fibromyalgia	Tramadol-Acetaminophen	315	FIQ, time lost from work, SF-36	8	3
13	Bradley et al. 2010 [18] + Arnold et al. 2009 [12] (Arnold et al. 2005 [11] + Chappell et al. 2008 [20])	Fibromyalgia	Duloxetine	1332	Bradley 2010 [18]: FIQ, Arnold 2009 [12]: SDS (Arnold 2005 [11]: BPI, Chappell 2008 [20]: SDS)	12-28	Review (Arnold et al. 2005 [11]: 5, Chappell et al. 2008 [20]: 3)
14	Straube et al. 2011 [4]	Fibromyalgia	Pregabalin	2757	FIQ, time lost from work, SF-36, SDS, MAF	8-14	Review
15	Chappell et al. 2009 [21]	Osteoarthritis (knee)	Duloxetine	231	BPI-I	13	5
16	Chappell et al. 2011 [22]	Osteoarthritis (knee)	Duloxetine	256	BPI-I	13	5
17	Markenson et al. 2005 [32]	Osteoarthritis	Oxycodone	109	BPI-I	12	4
18	Kavanaugh et al. 2006 [26]	Psoriatic arthritis	Infliximab	200	SF-36, employment status, employability, impact on productivity at work (VAS), time lost from work	22	3
19	Kavanaugh et al. 2013 [28]	Psoriatic arthritis	Golimumab	405	Impact on productivity at work (VAS)	24	4
20	Egsmose et al. 1997 [23]	Reactive arthritis	Sulphasalazine	83	Time lost from work	24	3
21	Bejarano et al. 2008 [15]	Rheumatoid arthritis	Adalimumab + MTX	148	Employment status, WIS, Time lost from work	56	5
22	Kavanaugh et al. 2009 [27]	Rheumatoid arthritis	Certolizumab pegol + MTX	1601	WPS, time lost from work	24 + 52	3
23	Meireles et al. 2010 [33]	Rheumatoid arthritis	Low-level laser therapy	82	DASH	8	5
24	Smolen et al. 2006 [37]	Rheumatoid arthritis	Infliximab + MTX	1004	Employability, time lost from work	54	5
25	Strand et al. 1999 [38]	Rheumatoid arthritis	Leflunomide, MTX	482	Productivity at work	48	4
26	Barkham et al. 2010 [13]	Ankylosing spondylitis	Entanercept	40	WIS, time lost from work	12	3
27	van der Heijde et al. 2006 [39]	Ankylosing spondylitis	Infliximab	279	SF-36, impact on productivity at work (VAS), time lost from work	24	4

BPI-I – Brief Pain Inventory: Interference with normal work; chr. – chronic; DASH – Disabilities of the Arm, Shoulder and Hand Questionnaire; FIQ – Fibromyalgia Impact Questionnaire; MAF – Multidimensional Assessment of Fatigue; MTX – methotrexate; OQS – Oxford Quality Scale; SDS – Sheehan Disability Scale; SF-36 – Short Form 36 Health Survey; TENS – transcutaneous electrical nerve stimulation; VAS – visual analogue scale; WIS – Work Instability Scale; WPAI – Work Productivity and Activity Impairment Questionnaire; WPS – Work Productivity Survey. For reviews, information in brackets refers to the primary publications on which the reviews were based.

The 31 publications reporting on work-related outcomes comprised only 0.23% of all publications on randomised placebo-controlled trials in chronic painful or rheumatic conditions (13,754 hits when searching as in Figure 1 but without the work-related search terms). The 31 studies that reported on work-related outcomes included a total of 11,434 patients (mean ages in the studies ranged from 34 to 63 years; overall 76% of patients were women). Among the 31 studies, the reporting of work-related outcomes was diverse: 16 different work-related outcomes were reported on (Table 1). The methodological study quality was generally high; only nine studies scored less than four (out of a maximum five) points on the OQS (Table 1).

Of 25 studies testing for the statistical significance of changes in work-related outcomes over the course of the trials, 14 (56%) reported a significant improvement; the others reported non-significant changes (Table 2).

**Table 2:** ‘Vote count’ of studies investigating work-related outcomes with statistical methods

**Significant improvement**	**Non-significant changes**
Bejarano et al. 2008 [15]	Albert et al. 2013 [10]
Bennett et al. 2003 [16]	Barkham et al. 2010 [13]
Bradley et al. 2010 [18]	Baron et al. 2010 [14]
Chappell et al. 2011 [22]	Chappell et al. 2009 [21]
He et al. 2005 [24]	Egsmose et al. 1997 [23]
Kavanaugh et al. 2006 [26]	Jarzem et al. 2005 [25]
Kavanaugh et al. 2009 [27]	Lehmann et al. 1986 [29]
Kavanaugh et al. 2013 [28]	Licciardone et al. 2003 [30]
Meireles et al. 2010 [33]	Skljarevski et al. 2009 [34]
Markenson et al. 2005 [32]	Straube et al. 2011 [4]
Skljarevski et al. 2010a [35]	van der Heijde et al. 2006 [39]
Skljarevski et al. 2010b [36]	
Smolen et al. 2006 [37]	
Strand et al. 1999 [38]	

The results of active treatment groups were pooled. Studies reported either significant improvements or non-significant changes; no study reported a significant worsening in work-related outcomes.

### Relationship between work and pain outcomes

**Figure 3:**
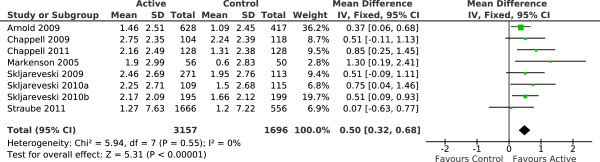
**Interference with work.** Meta-analysis of the improvement in interference with work over the duration of the studies. CI – confidence interval, Fixed – fixed effect model, IV – inverse variance, SD – standard deviation.

**Figure 4:**
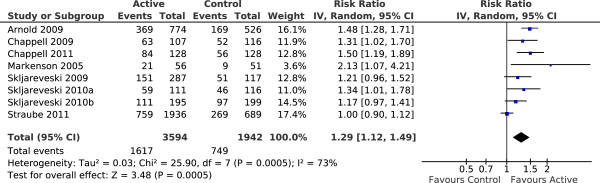
**30% pain responders.** Meta-analysis of the proportion of study participants attaining at least 30% reduction in pain intensity over the duration of the studies. CI – confidence interval, IV – inverse variance, Random – random effects model, SD – standard deviation.

We wanted to examine the relationship between work-related outcomes and pain-related outcomes. As elaborated above we primarily used responder outcomes for pain intensity and as regards work-related outcomes we used the outcome of ‘interference with work’ as estimated from the answers to similar questions about interference with work (or disruption of work) from three commonly used questionnaires. There were eight studies [4,12,21,22,32,34-36] that reported data on both interference with work and 30% pain responders; these studies were included in meta-analyses for the outcomes of interference with work (Figure 3) and 30% pain responders (Figure 4). For Straube et al. [4] data about pain responders was taken from Straube et al. [42].

These meta-analyses demonstrated statistically significant improvements in both outcomes with active therapy compared to placebo and also demonstrated that both outcomes behaved in a roughly similar manner, in individual studies and overall.

**Figure 5:**
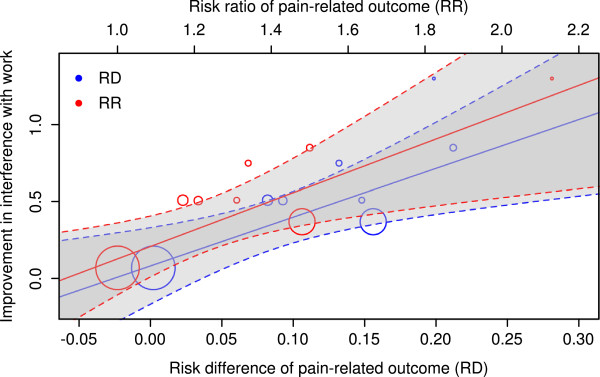
**Regression analysis.** Regression analysis of the improvement in interference with work from study beginning to end and ‘30% pain responders’, expressed as risk ratios [RR, red colour] or risk differences [RD, blue colour]. Interference with work was measured on a scale of 0–10 points. The size of the symbols represents the weights of the individual studies in the regression analysis (inverse variance). Regression lines are solid; broken lines mark the 95% confidence intervals.

To assess the degree of similarity between these outcomes formally, we performed regression analyses (Figure 5); these showed that the outcomes were significantly correlated when the 30% responder rates were expressed as risk differences (p = 0.012) as well as when they were expressed as risk ratios (p = 0.015). For the 50% responder rates we found a significant correlation when the responder rates were expressed as risk ratios (p = 0.038), but significance was narrowly missed when they were expressed as risk differences (p = 0.053). As the study of Skljarevski et al. [34] did not report 50% pain responder rates, it did not contribute to those analyses.

Nine studies reported mean differences of different pain-related and work-related outcomes. Six trials assessed interference with work and pain severity with questions from the Brief Pain Inventory [21,22,32,34-36]. Arnold et al. [12] reported data on disruption of work from the Sheehan Disability Scale and data on pain from the Brief Pain Inventory. Two studies used visual analogue scales to evaluate productivity at work and pain [26,39]. The data for the pain-related outcomes for these two studies, Kavanaugh et al. [26] and van der Heijde et al. [39], were taken from Antoni et al. [43] and van der Heijde et al. [44]. Because of the multiple outcomes reported across the papers we standardised the reported mean differences before performing linear regression. Again, a statistically significant correlation emerged (p = 0.035).

## Discussion

We were able to confirm our impression that work-related outcomes are indeed reported only very infrequently in chronic pain and rheumatology trials. This is somewhat surprising, given the importance of pain as a reason for decreased ability to work and given that a number of questionnaires that are commonly used in pain and rheumatology trials contain component questions addressing work-related outcomes [4]. The problem very likely is not one of the collection of work-related data but of the reporting of such data in publications.

This means two things. Firstly, that raising awareness of the importance of work-related outcomes among people and institutions involved in conducting trials in chronic pain and rheumatology is worthwhile and may lead to such data being reported more commonly for future trials and, secondly, that re-analysis of existing datasets could be informative, as long as access to work-related data can be obtained, preferably at the level of the individual patient.

Some limitations of our analysis need to be discussed: we based our systematic review on searching only one database and were limited (due to the linguistic skills of the authors, or lack thereof) to papers published in English or German. It is quite possible that we missed relevant studies published in journals not indexed in Medline, published in languages other than English or German, or published after our search had closed. We do not claim, therefore, to have identified all placebo-controlled trials conducted in chronic pain or rheumatologic diseases and reporting on work related outcomes. What we have, however, should be a fairly good and representative sample, including important trials published recently and in high impact journals.

We have confidence in our conclusion, therefore, that work-related outcomes are indeed reported very infrequently in journal publications resulting from chronic pain and rheumatology trials.

Chronic pain significantly interferes with work. A recent large systematic review of observational studies demonstrated the negative impact of chronic pain on work related outcomes [45]. It is therefore logical to suspect that pain relief will be associated with an improvement in the ability to work. Based on the evidence included in our systematic review (eight studies, with 5726 participants, conducted in fibromyalgia, back pain and osteoarthritis, investigating as treatments duloxetine, pregabalin, and oxicodone, and lasting eight to 28 weeks) we conclude that the outcome of improvement in interference with work is indeed closely related to the outcome of attaining at least 30% reduction in pain intensity over the course of the studies. Future work, in other painful conditions and assessing different treatments, needs to assess how robust this relationship is and whether pain-related outcomes (such as 30% pain responders) can perhaps be used to estimate interference with work. If they can, then this would open the door to using pain responder outcomes for evaluations of the impact of pain treatments also on work ability, which might be an important consideration for the assessment of pain treatments.

## Conclusions

Firstly, work-related outcomes are reported very infrequently, in substantially less than 1% of the publications we identified in our search for placebo-controlled trials in chronic painful and rheumatic diseases.

Secondly, work-related outcomes and pain responder outcomes are closely related. Future studies, perhaps based on individual patient analyses and meta-analyses of existing datasets, should address whether pain responder outcomes can be used to assess ability to work.

## Competing interests

SS has participated in grants from Pfizer and Reckitt-Benckiser and has received a lecture fee and an honorarium from Oxford Medical Knowledge for work on data from pain trials. TF is a consultant to Novartis Pharma AG, Biogen Idec, Phamalog and Grünenthal.

## Authors’ contributions

IW did the searching, data extraction, and study quality assessment, performed the analyses, and made the figures and tables. SS conceived of the study, supervised IW for his doctoral dissertation on this subject, and drafted the manuscript. TF provided statistical advice for the analysis and also supervised IW for his doctoral dissertation. All authors contributed to revising the manuscript and approved the final version of the manuscript.

## Supplementary Material

Additional file 1 **Excluded studies.** This file contains a table of excluded studies, with brief reasons for their exclusion and the references of the excluded studies. Click here for file
