# Supplementary material for: Work-related outcomes in randomised placebo-controlled pain trials: a systematic review and meta-analysis
Source: J Occup Med Toxicol. 2014 Jul 15;9:25. doi: 10.1186/1745-6673-9-25 (PMC4107475; doi:10.1186/1745-6673-9-25)
Supplement: Additional file 1 — Excluded studies. This file contains a table of excluded studies, with brief reasons for their exclusion and the references of the excluded studies. [file 1745-6673-9-25-S1.pdf]

Additional file 1

Excluded study	Reason for exclusion
Affaitati et al. 2009	Criteria for chronic pain were not met
Al-Smadi et al. 2003	No work-related data published
Arnold et al. 2011	No relevant work-related data published
Bagust et al. 2009	No work-related data published
Bennell et al. 2007	No work-related data published
Beurskens et al. 1997	Criteria for chronic pain were not met
Bjodal et al. 2008	Criteria for chronic pain were not met
Bombardier and Raboud 1991	No work-related data published
Borg et al. 1991	Not a placebo controlled trial
Braun et al. 2002	No work-related data published
Bresnihan 2002	The reported studies did not publish work-related data or were not placebo-controlled.
Brox et al. 2008	Not a placebo controlled trial
Bruyere et al. 2008	No work-related data published
Bunzli et al. 2011	The reported studies were not placebo-controlled or the criteria for chronic pain were not met.
Busanich andVerscheure 2006	No work-related data published
Cade et al. 1976	No data reported about the placebo-controlled phase
Cardiel et al. 2010	No work-related data published
Chen et al. 2006	No work-related data published
Chen et al. 2013	No work-related data published
Chiu et al. 2011	No work-related data published
Chou et al. 2007	Criteria for chronic pain were not met
Clare et al. 2004	Reported studies were not placebo-controlled
Clark et al. 2004	No work-related data published
Coghlan et al. 2008	The reported studies did not publish work-related data or were not placebo-controlled.
Cole et al. 2008	No relevant work-related data published
Davies et al. 1999	No work-related data published
Falco et al. 2012	No work-related data published
Fraser et al. 2005	No work-related data published
Freeman 2006	The reported studies were no randomised placebo-controlled trials or did not publish work-related data.
Furlan et al. 2008	The reported studies were not placebo-controlled or the criteria for chronic pain were not met.
Furlan et al. 2010	The work-related studies were not blinded.
Geisser et al. 2005	No work-related data published
Gibson and Waddell 2005	No work-related data published
Goldenberg et al. 1996	No work-related data published

Han et al. 2008	No comparison between active treatment and control
Hennigan et al. 2008	No work-related data published
Hewitson et al. 2000	No work-related data published
Heymans et al. 2004	Not a placebo controlled trial
Hill and Hill 1975	No work-related data published
Hudson et al. 2009	No relevant work-related data published
Hunter et al. 2009	No work-related data published
Hutchinson et al. 2012	The work-related study was not placebo-controlled.
Itoh and Kitakoji 2007	No work-related data published
Iversen et al. 2011	No work-related data published
Kay and Rahman 2010	No work-related data published
Khadilkar et al. 2008	The only work-related study reported was Jarzem et al. 2005 (which is included in the review). No further work-related data were reported.
Kimel et al. 2011	No work-related data published
Kirveskari and Alanen 1984	Criteria for chronic pain were not met
Kuijpers et al. 2011	No work-related data published
Lerner et al. 2012	No relevant work-related data published
Listing et al. 2004	Not a placebo controlled trial
Malottki et al. 2011	No work-related data published
Manheimer et al. 2005	The reported studies were not placebo-controlled or they did not contain work-related data.
McLeod et al. 2007	No work-related data published
Mittendorf et al. 2008	Not a placebo controlled trial
Moreland 2004	The reported studies did not contain work-related data or were not placebo-controlled.
Navarro-Millán et al. 2012	No work-related data published
Niemistö et al. 2005	Not a placebo controlled trial
Nogid and Pham 2006	No work-related data published
O'Connell et al. 2011	No work-related data published
Osterhaus et al. 2009	No comparison between active treatment and control
Pach et al. 2011	No work-related data published
Pope et al. 2004	No work-related data published
Price et al. 2005	Criteria for chronic pain were not met
Rodgers et al. 2011	No work-related data published
Rogvi-Hansen et al. 1991	Criteria for chronic pain were not met
Rossini et al. 2007	No work-related data published
Rubinstein et al. 2011	The reported studies did not contain work-related data or the criteria for chronic pain were not met.
Ruiz Garcia et al. 2011	No work-related data published
Schmitz et al. 2012	No work-related data published
Schneider et al. 2012	No work-related data published
Scott 1999	No work-related data published
Skljarevski et al. 2010c	Open lable extension of the study: Skljarevski et al. 2010a (which is included in the review)
Skljarevski et al. 2011	No work-related data published
Skljarevski et al. 2012	No work-related data published
Sörensen et al. 1996	Not a placebo controlled trial

Stacey and Glanzman 2003	No work-related data published
Szczurko et al. 2009	Criteria for chronic pain were not met
Thompson et al. 1988	No work-related data published
Turner et al. 1995	No randomised, clinical study reported
Wernicke et al. 2007	No work-related data published
Winemiller et al. 2005	Criteria for chronic pain were not met
Woolacott et al. 2006	No work-related data published
Yang et al. 2012	No work-related data published
Zanette et al. 2008	No work-related data published

References

1. Affaitati G, Fabrizio A, Savini A, Lerza R, Tafuri E, Costantini R, Lapenna D, Giamberardino MA: **A randomized, controlled study comparing a lidocaine patch, a placebo patch, and anesthetic injection for treatment of trigger points in patients with myofascial pain syndrome: evaluation of pain and somatic pain thresholds.** *Clin Ther* 2009, **31**:705-720.

2. Al-Smadi J, Warke K, Wilson I, Cramp AF, Noble G, Walsh DM, Lowe-Strong AS: **A pilot investigation of the hypoalgesic effects of transcutaneous electrical nerve stimulation upon low back pain in people with multiple sclerosis.** *Clin Rehabil* 2003, **17:**742-749.

3. Arnold LM, Zlateva G, Sadosky A, Emir B, Whalen E: **Correlations between fibromyalgia symptom and function domains and patient global impression of change: a pooled analysis of three randomized, placebo-controlled trials of pregabalin.** *Pain Med* 2011, **12**:260-267.

4. Bagust A, Boland A, Hockenhull J, Fleeman N, Greenhalgh J, Dundar Y, Proudlove C, Kennedy T, Moots R, Williamson P, Dickson R: **Rituximab for the treatment of rheumatoid arthritis.** *Health Technol Assess* 2009, **13 Suppl 2**:23-29.

5. Bennell K, Coburn S, Wee E, Green S, Harris A, Forbes A, Buchbinder R: **Efficacy and cost-effectiveness of a physiotherapy program for chronic rotator cuff pathology: a protocol for a randomised, double-blind, placebo-controlled trial.** *BMC Musculoskelet Disord* 2007, **8**:86.

6. Beurskens AJ, de Vet HC, Köke AJ, Regtop W, van der Heijden GJ, Lindeman E, Knipschild PG: **Efficacy of traction for nonspecific low back pain. 12-week and 6-month results of a randomized clinical trial.** *Spine (Phila Pa 1976)* 1997, **22**:2756-2762.

7. Bjordal JM, Lopes-Martins RA, Joensen J, Couppe C, Ljunggren AE, Stergioulas A, Johnson MI: **A systematic review with procedural assessments and meta-analysis of low level laser therapy in lateral elbow tendinopathy (tennis elbow).** *BMC Musculoskelet Disord* 2008, **9**:75.

8. Bombardier C, Raboud J: **A comparison of health-related quality-of-life measures for rheumatoid arthritis research. The Auranofin Cooperating Group.** *Control Clin Trials* 1991, **12**:243S-256S.

9. Borg G, Allander E, Berg E, Brodin U, From A, Trang L: **Auranofin treatment in early rheumatoid arthritis may postpone early retirement. Results from a 2-year double blind trial.** *J Rheumatol.* 1991, **18**:1015-1020.

10. Braun J, Brandt J, Listing J, Zink A, Alten R, Golder W, Gromnica-Ihle E, Kellner H, Krause A, Schneider M, Sörensen H, Zeidler H, Thriene W, Sieper J: **Treatment of active ankylosing spondylitis with infliximab: a randomised controlled multicentre trial.** *Lancet* 2002, **359**:1187-1193.

11. Bresnihan B: **Anakinra as a new therapeutic option in rheumatoid arthritis: clinical results and perspectives.** *Clin Exp Rheumatol* 2002, **20**:S32-S34.

12. Brox JI, Storheim K, Grotle M, Tveito TH, Indahl A, Eriksen HR: **Systematic review of back schools, brief education, and fear-avoidance training for chronic low back pain.** *Spine J* 2008, **8**:948-958.

13. Bruyere O, Delferriere D, Roux C, Wark JD, Spector T, Devogelaer JP, Brixen K, Adami S, Fechtenbaum J, Kolta S, Reginster JY: **Effects of strontium ranelate on spinal osteoarthritis progression.** *Ann Rheum Dis* 2008, **67**:335-339.

14. Bunzli S, Gillham D, Esterman A: **Physiotherapy-provided operant conditioning in the management of low back pain disability: A systematic review.** *Physiother Res Int* 2011, **16**:4-19.

15. Busanich BM, Verscheure SD: **Does McKenzie therapy improve outcomes for back pain?** *J Athl Train* 2006, **41**:117-119.

16. Cade R, Stein G, Pickering M, Schlein E, Spooner G: **Low dose, long-term treatment of rheumatoid arthritis with azathioprine.** *South Med J* 1976, **69**:388-392.

17. Cardiel MH, Tak PP, Bensen W, Burch FX, Forejtova S, Badurski JE, Kakkar T, Bevirt T, Ni L, McCroskery E, Jahreis A, Zack DJ: **A phase 2 randomized, double-blind study of AMG 108, a fully human monoclonal antibody to IL-1R, in patients with rheumatoid arthritis.** *Arthritis Res Ther* 2010, **12**:R192.

18. Chen J, Veras MM, Liu C, Lin J: **Methotrexate for ankylosing spondylitis.** *Cochrane Database Syst Rev* 2013, **2**:CD004524.

19. Chen YF, Jobanputra P, Barton P, Jowett S, Bryan S, Clark W, Fry-Smith A, Burls A: **A systematic review of the effectiveness of adalimumab, etanercept and infliximab for the treatment of rheumatoid arthritis in adults and an economic evaluation of their cost-effectiveness.** *Health Technol Assess* 2006, **10**:42.

20. Chiu CK, Low TH, Tey YS, Singh VA, Shong HK: **The efficacy and safety of intramuscular injections of methylcobalamin in patients with chronic nonspecific low**

back pain: a randomised controlled trial. *Singapore Med J* 2011, **52**:868-873.

21. Chou R, Huffman LH; American Pain Society; American College of Physicians: **Nonpharmacologic therapies for acute and chronic low back pain: a review of the evidence for an American Pain Society/American College of Physicians clinical practice guideline.** *Ann Intern Med* 2007, **147**:492-504.

22. Clare HA, Adams R, Maher CG: **A systematic review of efficacy of McKenzie therapy for spinal pain.** *Aust J Physiother* 2004, **50**:209-216.

23. Clark W, Jobanputra P, Barton P, Burls A: **The clinical and cost-effectiveness of anakinra for the treatment of rheumatoid arthritis in adults: a systematic review and economic analysis.** *Health Technol Assess* 2004, **8**:18.

24. Coghlan JA, Buchbinder R, Green S, Johnston RV, Bell SN: **Surgery for rotator cuff disease.** *Cochrane Database Syst Rev* 2008, **1**:CD005619.

25. Cole JC, Li T, Lin P, MacLean R, Wallenstein GV: **Treatment impact on estimated medical expenditure and job loss likelihood in rheumatoid arthritis: re-examining quality of life outcomes from a randomized placebo-controlled clinical trial with abatacept.** *Rheumatology* 2008, **47**:1044-1050.

26. Davies GM, Watson DJ, Bellamy N: **Comparison of the responsiveness and relative effect size of the western Ontario and McMaster Universities Osteoarthritis Index and the short-form Medical Outcomes Study Survey in a randomized, clinical trial of osteoarthritis patients.** *Arthritis Care Res* 1999, **12**:172-179.

27. Falco FJ, Manchikanti L, Datta S, Wargo BW, Geffert S, Bryce DA, Atluri S, Singh V, Benyamin RM, Sehgal N, Ward SP, Helm S 2nd, Gupta S, Boswell MV: **Systematic review of the therapeutic effectiveness of cervical facet joint interventions: an update.** *Pain Physicia* 2012, **15**:E839-E868.

28. Fraser AD, van Kuijk AW, Westhovens R, Karim Z, Wakefield R, Gerards AH, Landewé R, Steinfeld SD, Emery P, Dijkmans BA, Veale DJ: **A randomised, double blind, placebo controlled, multicentre trial of combination therapy with methotrexate plus ciclosporin in patients with active psoriatic arthritis.** *Ann Rheum Dis* 2005, **64**:859-864.

29. Freeman BJ: **IDET: a critical appraisal of the evidence.** *Eur Spine J* 2006, **15**:S448-S457.

30. Furlan AD, Imamura M, Dryden T, Irvin E: **Massage for low back pain: an updated systematic review within the framework of the Cochrane Back Review Group.** *Spine (Phila Pa 1976)* 2009, **34**:1669-1684.

31. Furlan AD, Yazdi F, Tsertsvadze A, Gross A, Van Tulder M, Santaguida L, Cherkin D, Gagnier J, Ammendolia C, Ansari MT, Ostermann T, Dryden T, Doucette S, Skidmore B, Daniel R, Tsouros S, Weeks L, Galipeau J: **Complementary and alternative therapies for back pain II.** *AHRQ Publication* 2010, **10**:E007.

32. Geisser ME, Wiggert EA, Haig AJ, Colwell MO: **A randomized, controlled trial of**

manual therapy and specific adjuvant exercise for chronic low back pain. *Clin J Pain* 2005, **21**:463-470.

33. Gibson JN, Waddell G: **Surgery for degenerative lumbar spondylosis.** *Cochrane Database Syst Rev* 2005, **4**:CD001352.

34. Goldenberg D, Mayskiy M, Mossey C, Ruthazer R, Schmid C: **A randomized, double-blind crossover trial of fluoxetine and amitriptyline in the treatment of fibromyalgia.** *Arthritis Rheum* 1996, **39**:1852-1859.

35. Han C, Smolen J, Kavanaugh A, St Clair EW, Baker D, Bala M: **Comparison of employability outcomes among patients with early or long-standing rheumatoid arthritis.** *Arthritis Rheum* 2008, **59**:510-514.

36. Hennigan S, Ackermann C, Kavanaugh A: Hennigan S1, Ackermann C, Kavanaugh A: **Adalimumab in ankylosing spondylitis: an evidence-based review of its place in therapy.** *Core Evid* 2008, **2**:295-305.

37. Hewitson PJ, Debroe S, McBride A, Milne R: **Leflunomide and rheumatoid arthritis: a systematic review of effectiveness, safety and cost implications.** *J Clin Pharm Ther* 2000, **25**:295-302.

38. Heymans MW, van Tulder MW, Esmail R, Bombardier C, Koes BW: **Back schools for non-specific low-back pain.** *Cochrane Database Syst Rev* 2004, **4**:CD000261.

39. Hill HF, Hill AG: **Ankylosing spondylitis: open long-term and double-blind crossover studies with naproxen.** *J Clin Pharmacol* 1975, **15**:355-362.

40. Hudson JI, Arnold LM, Bradley LA, Choy EH, Mease PJ, Wang F, Ahl J, Wohlreich MM: **What makes patients with fibromyalgia feel better? Correlations between Patient Global Impression of Improvement and changes in clinical symptoms and function: a pooled analysis of 4 randomized placebo-controlled trials of duloxetine.** *J Rheumatol* 2009, **36**:2517-2522.

41. Hunter AM, Leuchter AF, Cook IA, Abrams M, Siegman BE, Furst DE, Chappell AS: **Brain functional changes and duloxetine treatment response in fibromyalgia: a pilot study.** *Pain Med* 2009, **10**:730-738.

42. Hutchinson AJ, Ball S, Andrews JC, Jones GG: **The effectiveness of acupuncture in treating chronic non-specific low back pain: a systematic review of the literature.** *J Orthop Surg Res* 2012, **7**:36.

43. Itoh K, Kitakoji H: **Acupuncture for chronic pain in Japan: a review.** *Evid Based Complement Alternat Med* 2007, **4**:431-438.

44. Iversen T, Solberg TK, Romner B, Wilsgaard T, Twisk J, Anke A, Nygaard O, Hasvold T, Ingebrigtsen T: **Effect of caudal epidural steroid or saline injection in chronic lumbar radiculopathy: multicentre, blinded, randomised controlled trial.** *BMJ* 2011, **343**:d5278.

45. Kay J, Rahman MU: **Golimumab: A novel human anti-TNF-alpha monoclonal antibody for the treatment of rheumatoid arthritis, ankylosing spondylitis, and psoriatic arthritis.** *Core Evid* 2010, **4**:159-170.

46. Khadilkar A, Odebiyi DO, Brosseau L, Wells GA: **Transcutaneous electrical nerve stimulation (TENS) versus placebo for chronic low-back pain.** *Cochrane Database Syst Rev* 2008, **4**:CD003008.

47. Kimel M, Revicki D, Rao S, Fryback D, Feeny D, Harnam N, Thompson C, Cifaldi M: **Norms-based assessment of patient-reported outcomes associated with adalimumab monotherapy in patients with ankylosing spondylitis.** *Clin Exp Rheumatol* 2011, **29**:624-632.

48. Kirveskari P, Alanen P: **Effect of occlusal treatment on sick leaves in TMJ dysfunction patients with head and neck symptoms.** *Community Dent Oral Epidemiol* 1984, **12**:78-81.

49. Kuijpers T, van Middelkoop M, Rubinstein SM, Ostelo R, Verhagen A, Koes BW, van Tulder MW: **A systematic review on the effectiveness of pharmacological interventions for chronic non-specific low-back pain.** *Eur Spine J* 2011, **20**:40-50.

50. Lerner D, Chang H, Rogers WH, Benson C, Chow W, Kim MS, Biondi D: **Imputing at-work productivity loss using results of a randomized controlled trial comparing tapentadol extended release and oxycodone controlled release for osteoarthritis pain.** *J Occup Environ Med* 2012, **54**:933-938.

51. Listing J, Brandt J, Rudwaleit M, Zink A, Sieper J, Braun J: **Impact of anti-tumour necrosis factor alpha treatment on admissions to hospital and days of sick leave in patients with ankylosing spondylitis.** *Ann Rheum Dis* 2004, **63**:1670-1672.

52. Malottki K, Barton P, Tsourapas A, Uthman AO, Liu Z, Routh K, Connock M, Jobanputra P, Moore D, Fry-Smith A, Chen YF: **Adalimumab, etanercept, infliximab, rituximab and abatacept for the treatment of rheumatoid arthritis after the failure of a tumour necrosis factor inhibitor: a systematic review and economic evaluation.** *Health Technol Assess* 2011, **15**:14.

53. Manheimer E, White A, Berman B, Forys K, Ernst E: **Meta-analysis: acupuncture for low back pain.** *Ann Intern Med* 2005, **142**:651-663.

54. McLeod C, Bagust A, Boland A, Dagenais P, Dickson R, Dundar Y, Hill RA, Jones A, Mujica Mota R, Walley T: **Adalimumab, etanercept and infliximab for the treatment of ankylosing spondylitis: a systematic review and economic evaluation.** *Health Technol Assess* 2007, **11**:28.

55. Mittendorf T, Dietz B, Sterz R, Cifaldi MA, Kupper H, von der Schulenburg JM: **Personal and economic burden of late-stage rheumatoid arthritis among patients treated with adalimumab: an evaluation from a patient's perspective.** *Rheumatology (Oxford)* 2008, **47**:188-193.

56. Moreland LW: **Drugs that block tumour necrosis factor: experience in patients with rheumatoid arthritis.** *Pharmacoeconomics* 2004, **22 (2 Suppl 1)**:39-53.

57. Navarro-Millán I, Singh JA, Curtis JR: **Systematic review of tocilizumab for rheumatoid arthritis: a new biologic agent targeting the interleukin-6 receptor.** Clin Ther 2012, **34**:788-802.

58. Niemistö L, Rissanen P, Sarna S, Lahtinen-Suopanki T, Lindgren KA, Hurri H: **Cost-effectiveness of combined manipulation, stabilizing exercises, and physician consultation compared to physician consultation alone for chronic low back pain: a prospective randomized trial with 2-year follow-up.** *Spine (Phila Pa 1976)* 2005, **30**:1109-1115.

59. Nogid A, Pham DQ: **Role of abatacept in the management of rheumatoid arthritis.** *Clin Ther* 2006, **28**:1764-1778.

60. O'Connell NE, Wand BM, Marston L, Spencer S, Desouza LH: **Non-invasive brain stimulation techniques for chronic pain. A report of a Cochrane systematic review and meta-analysis.** *Eur J Phys Rehabil Med* 2011, **47**:309-326.

61. Osterhaus JT, Purcaru O, Richard L: **Discriminant validity, responsiveness and reliability of the rheumatoid arthritis-specific Work Productivity Survey (WPS-RA).** *Arthritis Res Ther* 2009, **11**:R73.

62. Pach D, Brinkhaus B, Roll S, Wegscheider K, Icke K, Willich SN, Witt CM: **Efficacy of injections with Disci/Rhus toxicodendron compositum for chronic low back pain--a randomized placebo-controlled trial.** *PLoS One* 2011, **6**:e26166.

63. Pope JE, Prashker M, Anderson J: **The efficacy and cost effectiveness of N of 1 studies with diclofenac compared to standard treatment with nonsteroidal antiinflammatory drugs in osteoarthritis.** *J Rheumatol* 2004, **31**:140-149.

64. Price C, Arden N, Coglan L, Rogers P: **Cost-effectiveness and safety of epidural steroids in the management of sciatica.** *Health Technol Assess* 2005, **9**:33.

65. Rodgers M, Epstein D, Bojke L, Yang H, Craig D, Fonseca T, Myers L, Bruce I, Chalmers R, Bujkiewicz S, Lai M, Cooper N, Abrams K, Spiegelhalter D, Sutton A, Sculpher M, Woolacott N: **Etanercept, infliximab and adalimumab for the treatment of psoriatic arthritis: a systematic review and economic evaluation.** *Health Technol Assess* 2011, **15**:10.

66. Rogvi-Hansen B, Ellitsgaard N, Funch M, Dall-Jensen M, Prieske J: **Low level laser treatment of chondromalacia patellae.** *Int Orthop* 1991, **15**:359-361.

67. Rossini M, Di Munno O, Valentini G, Bianchi G, Biasi G, Cacace E, Malesci D, La Montagna G, Viapiana O, Adami S: **Double-blind, multicenter trial comparing acetyl l-carnitine with placebo in the treatment of fibromyalgia patients.** *Clin Exp Rheumatol* 2007, **25**:182-188.

68. Rubinstein SM, van Middelkoop M, Assendelft WJ, de Boer MR, van Tulder MW: **Spinal manipulative therapy for chronic low-back pain: an update of a Cochrane review.** *Spine (Phila Pa 1976)* 2011, **36**:E825-E846.

69. Ruiz Garcia V, Jobanputra P, Burls A, Cabello JB, Gálvez Muñoz JG, Saiz Cuenca ES, Fry-Smith A: **Certolizumab pegol (CDP870) for rheumatoid arthritis in adults.** *Cochrane Database Syst Rev* 2011, **2**:CD007649.

70. Schmitz S, Adams R, Walsh CD, Barry M, FitzGerald O: **A mixed treatment comparison of the efficacy of anti-TNF agents in rheumatoid arthritis for methotrexate non-responders demonstrates differences between treatments: a Bayesian approach.** *Ann Rheum Dis* 2012, **71**:225-230.

71. Schneider H, Maheu E, Cucherat M: **Symptom-modifying effect of chondroitin sulfate in knee osteoarthritis: a meta-analysis of randomized placebo-controlled trials performed with structum(®).** *Open Rheumatol J* 2012, **6**:183-189.

72. Scott DL: **Leflunomide improves quality of life in rheumatoid arthritis.** *Scand J Rheumatol Suppl.* 1999, **112**:23-29.

73. Skljarevski V, Liu P, Zhang S, Ahl J, Martinez JM: **Efficacy and safety of duloxetine in patients with chronic low back pain who Uused versus did not use concomitant nonsteroidal anti-inflammatory drugs or acetaminophen: A post hoc pooled analysis of 2 randomized, placebo-controlled rrials.** *Pain Res Treat* 2012, **2012**:296710.

74. Skljarevski V, Zhang S, Chappell AS, Walker DJ, Murray I, Backonja M: **Maintenance of effect of duloxetine in patients with chronic low back pain: a 41-week uncontrolled, dose-blinded study.** *Pain Med* 2010, **11**:648-657.

75. Skljarevski V, Zhang S, Iyengar S, D'Souza D, Alaka K, Chappell A, Wernicke J: **Efficacy of Duloxetine in Patients with Chronic Pain Conditions.** *Curr Drug Ther* 2011, **6**:296-303.

76. Sörensen J, Aaro S, Bengtsson M, Kalman S, Reigo T, Tropp H: **Can a pharmacological pain analysis in patients with chronic low back pain predict the outcome of lumbar fusion? Preliminary report.** *Eur Spine J* 1996, **5**:326-331.

77. Stacey BR, Glanzman RL: **Use of gabapentin for postherpetic neuralgia: results of two randomized, placebo-controlled studies.** *Clin Ther* 2003, **25**:2597-608.

78. Szczurko O, Cooley K, Mills EJ, Zhou Q, Perri D, Seely D: Szczurko O1, Cooley K, Mills EJ, Zhou Q, Perri D, Seely D: **Naturopathic treatment of rotator cuff tendinitis among Canadian postal workers: a randomized controlled trial.** *Arthritis Rheum* 2009, **61**:1037-1045.

79. Thompson MS, Read JL, Hutchings HC, Paterson M, Harris ED Jr: **The cost effectiveness of auranofin: results of a randomized clinical trial.** *J Rheumatol* 1988, **15**:35-42.

80. Turner JA, Loeser JD, Bell KG: **Spinal cord stimulation for chronic low back pain: a**

systematic literature synthesis. *Neurosurgery* 1995, **37**:1088-1095.

81. Wernicke JF, Wang F, Pritchett YL, Smith TR, Raskin J, D'Souza DN, Iyengar S, Chappell AS: **An open-label 52-week clinical extension comparing duloxetine with routine care in patients with diabetic peripheral neuropathic pain.** *Pain Med* 2007, **8**:503-513.

82. Winemiller MH, Billow RG, Laskowski ER, Harmsen WS: **Effect of magnetic vs sham-magnetic insoles on nonspecific foot pain in the workplace: a randomized, double-blind, placebo-controlled trial.** *Mayo Clin Proc* 2005, **80**:1138-1145.

83. Woolacott N, Bravo Vergel Y, Hawkins N, Kainth A, Khadjesari Z, Misso K, Light K, Asseburg C, Palmer S, Claxton K, Bruce I, Sculpher M, Riemsma R: **Etanercept and infliximab for the treatment of psoriatic arthritis: a systematic review and economic evaluation.** *Health Technol Assess* 2006, **10**:31.

84. Yang H, Craig D, Epstein D, Bojke L, Light K, Bruce IN, Sculpher M, Woolacott N: **Golimumab for the treatment of psoriatic arthritis: a NICE single technology appraisal.** *Pharmacoeconomics* 2012, **30**:257-270.

85. Zanette Sde A, Born IG, Brenol JC, Xavier RM: **A pilot study of acupuncture as adjunctive treatment of rheumatoid arthritis.** *Clin Rheumatol* 2008, **27**:627-635.
